# Comparative Proteomic Analysis of *Paulownia fortunei* Response to Phytoplasma Infection with Dimethyl Sulfate Treatment

**DOI:** 10.1155/2017/6542075

**Published:** 2017-09-05

**Authors:** Zhen Wei, Zhe Wang, Xiaoyu Li, Zhenli Zhao, Minjie Deng, Yanpeng Dong, Xibing Cao, Guoqiang Fan

**Affiliations:** ^1^Institute of Paulownia, Henan Agricultural University, Zhengzhou, Henan 450002, China; ^2^College of Forestry, Henan Agricultural University, Zhengzhou, Henan 450002, China

## Abstract

*Paulownia fortunei* is a widely cultivated economic forest tree species that is susceptible to infection with phytoplasma, resulting in Paulownia witches' broom (PaWB) disease. Diseased *P. fortunei* is characterized by stunted growth, witches' broom, shortened internodes, and etiolated and smaller leaves. To understand the molecular mechanism of its pathogenesis, we applied isobaric tags for relative and absolute quantitation (iTRAQ) and liquid chromatography coupled with tandem mass spectrometry approaches to study changes in the proteomes of healthy *P. fortunei*, PaWB-infected *P. fortunei*, and PaWB-infected *P. fortunei* treated with 15 mg·L^−1^ or 75 mg·L^−1^ dimethyl sulfate. We identified 2969 proteins and 104 and 32 differentially abundant proteins that were phytoplasma infection responsive and dimethyl sulfate responsive, respectively. Based on our analysis of the different proteomes, 27 PaWB-related proteins were identified. The protein-protein interactions of these 27 proteins were analyzed and classified into four groups (photosynthesis-related, energy-related, ribosome-related, and individual proteins). These PaWB-related proteins may help in developing a deeper understanding of how PaWB affects the morphological characteristics of *P. fortunei* and further establish the mechanisms involved in the response of *P. fortunei* to phytoplasma.

## 1. Introduction

*Paulownia fortunei* is a fast-growing tree species native to China, which belongs to the Scrophulariaceae family. It is planted widely for its high economic value and medicinal properties. Unfortunately, it is susceptible to Paulownia witches' broom disease, a serious and destructive disease, caused by phytoplasmas that belong to “*Candidatus* Phytoplasma australiense.” These phytoplasmas are unculturable, lack cell walls, and have obligated symbiotic relationships with insects and plants [1], resulting in symptoms of witches' broom, short internodes, phyllody, and yellowing of leaves, eventually resulting in death [2, 3]. Phytoplasmas infect *P. fortunei* plants through phloem-feeding insect vectors and then reproduce and transmit in phloem sieve cells to obtain nutrients and carbohydrates as well as secreting effectors.

Phytoplasmas have been determined to infect more than 100 plant species, and intensive studies have been initiated to research the interaction between phytoplasmas and host plants at the molecular level. Mulberry is an economic woody plant that can be invaded by phytoplasmas resulting in yellow dwarf disease. In a previous 2-DE study in mulberry, 37 differentially abundant proteins (DAPs) were detected, and the abundance of some proteins that were found to be indispensable for photosynthesis was reduced in infected mulberry leaves [4]. In subsequent small RNA and metabolomic analyses in infected mulberry, phytoplasma was thought to disturb the balance of phytohormones as well as to promote the presence of hydrogen peroxide and superoxide [5, 6]. Phytoplasmas also infect *Catharanthus roseus* resulting in peanut witches' broom. The microRNA (miRNA) miR396 was shown to be inhibited by *PHYL1* effector, thereby promoting the expression of its target gene *SHORT VEGETATIVE PHASE* (SVP) in infected *C. roseus* [7]. Genes involved in defense and flowering in *C. roseus* were found by transcriptome analysis to be altered. For example, the expression levels of *CrSVP1/2* and *CrFT* increased, which played critical roles particularly in leafy flower transition [8]. Phytoplasmas have also been studied in other plant species, such as lime tree and grapevine [9, 10].

Many researchers, including members of our group, have studied Paulownia witches' broom (PaWB) for a number of decades. Recently, we found that dimethyl sulfate (DMS) could make the morphology of PaWB-infected seedling become normal [11]. In addition, studies of PaWB at the physiological, biochemical, and molecular levels have been performed using transcriptome, miRNA, and degradome analyses and methylation-sensitive amplified polymorphism approaches [12–17]. As a result, some genes and miRNAs, possibly associated with the response to phytoplasma invasion, have been identified in Paulownia. For instance, Mou et al. found that the expression levels of genes associated with cytokinin biosynthesis and cell wall were raised, while the expression levels of genes involved in photosynthesis were reduced in infected compared with healthy Paulownia [16]. We verified that the expression of genes related to phytohormones, phenylpropanoid, and circadian rhythm were changed in phytoplasma-infected *P. fortunei* [18]. However, the molecular mechanisms of PaWB are still poorly understood.

Proteins play important roles in metabolism, catalyzing many biochemical reactions involved in the lifecycles of plants [19]. Proteomics, the simultaneous large-scale analysis of the protein component of an organism, is considered to provide more useful information than genomics and has been applied successfully to gain insights into plant defense responses to pathogens [19]. Protein labeling by isobaric tags for relative and absolute quantitation (iTRAQ) for mass spectrometry has wide application in proteomics and has several advantages over traditional technologies like 2-DE [20–23]. Firstly, all types of proteins can be labeled by iTRAQ, which facilitates reliability and coverage of protein identification. Secondly, iTRAQ is easy to perform and can be combined directly with mass spectrometry. Thirdly, eight samples can be compared at the same time by iTRAQ, which increases label efficiency and reduces technical error.

In this study, we applied proteomic analysis to investigate alterations in protein abundance levels before and after phytoplasma invasion in Paulownia. We used healthy *P. fortunei* (PF), PaWB-infected *P. fortunei* (PFI), and PFI treated with 15 mg·L^−1^ or 75 mg·L^−1^ DMS to explore the interactions between phytoplasma and *P. fortunei* by iTRAQ and liquid chromatography coupled with tandem mass spectrometry (LC-MS/MS). The proteomic data analysis and functional annotations of the proteins detected in our study showed that the phytoplasma was highly associated with mechanisms related to antioxidants and phytohormones in *P. fortunei*. Our results will provide a foundation for better understanding the mechanisms of phytoplasma infection in plants in the future.

## 2. Materials and Methods

### 2.1. Plant Materials and Treatments

The *P. fortunei* seedlings used in this study were obtained from the Institute of Paulownia, Henan Agricultural University, China. Seedlings of healthy *P. fortunei* and *P. fortunei* infected with PaWB were cultured on 1/2 Murashige-Skoog (MS) medium with 20 g·L^−1^ sucrose and 8 g·L^−1^ agar for 30 days. Then, the 4 cm long terminal buds of the PF, PFI, and PFI with 15 mg·L^−1^ (PFI-15) and 75 mg·L^−1^ (PFI-75) DMS were soaked in 100 mL flasks at 16°C under dark. After soaking for 5 hours, the seedlings were cultured on 1/2 MS medium with 20 g·L^−1^ sucrose and 8 g·L^−1^ agar in 100 mL flasks at 25 ± 2°C under 130 *μ*mol·m^−2^·s^−1^ intensity light for 16 h every day. After 30 days, the 1.5 cm long terminal buds of the seedlings were sheared and immediately stored at −80°C for further study. The terminal buds from three individual plants were combined to form one biological replicate, and at least three biological replicates were used for each treatment.

### 2.2. Phytoplasma Detection

We applied nested PCR to detect phytoplasma in seedlings according to the method described by Zhao et al. The primer pairs used to amplify the 16S rDNA of the phytoplasma were R16mF1/R16mR1 (5′-CATGCAAGTCGAACGGA-3′/5′-CTTAACCCCAATCATCGAC-3′) and R16mF2/R16mR2 (5′-ACGACTGCTAAGACTGG-3′/5′-CGGGGTTTGTACACACCGC-3′). Agarose gel electrophoresis was applied as described by Fan et al. [24]. Three biological replicates were performed.

### 2.3. Protein Extraction

The four stored *P. fortunei* samples (PF, PFI, PFI-15, and PFI-75) were ground to powder in liquid nitrogen and then extracted with lysis buffer (7 M Urea, 2 M Thiourea, 4% CHAPS, 40 mM Tris-HCl, and pH 8.5) containing 1 mM PMSF and 2 mM EDTA. The proteins were extracted, reduced, and alkylated according to a previously described procedure [25]. The protein was quantified using the Bradford method [26]. Finally, the quantified proteins in the supernatant were extracted and stored at −80°C prior to sample cleanup if not for immediate use. Two replicates were used for each accession.

### 2.4. Protein Digestion, iTRAQ Labeling, and Strong Cation Exchange

Total protein (100 *μ*g) from each sample solution was digested with Trypsin Gold (Promega, Madison, WI, USA; protein : trypsin ratio of 30 : 1) at 37°C for 16 hours [27]. After trypsin digestion, the peptides were dried by vacuum centrifugation. Peptides were reconstituted in 0.5 M TEAB and processed according to the manufacturer's protocol for the 8-plex iTRAQ reagent (Applied Biosystems, Foster City, CA, USA). The iTRAQ experiment was performed on two independent biological replicates. Strong cation exchange chromatography was performed with a LC-20AB HPLC Pump system, as described by Dong et al. [28]. The procedure used for the strong cation exchange chromatography is described in Supplemental Protocol 1 available online at https://doi.org/10.1155/2017/6542075.

### 2.5. LC-MS/MS Analysis

The mass spectroscopy analysis was performed using a TripleTOF 5600 mass spectrometer (AB SCIEX, Framingham, MA, USA) and coupled with an online micro flow HPLC system, as described previously [29]. Details are given in Supplemental Protocol 2.

### 2.6. Proteomic Data Analysis

The mass spectrometry data were processed using Proteome Discoverer 1.2 software (Thermo Fisher Scientific, San Jose, CA, USA). Details are given in Supplemental Protocol 3. Protein identification and quantification were performed using the Mascot search engine (Matrix Science, London, UK; version 2.3.02) against the NCBI plant database, which contained 1,495,258 sequences. For protein identification, only peptides with significance scores (≥20) at the 99% confidence interval obtained by a Mascot probability analysis greater than “identity” were counted as identified. Every confident protein identification was associated with at least one unique peptide. For protein quantitation, a protein had to contain at least two unique peptides. The quantitative protein ratios were weighted and normalized by the median ratio in Mascot. Proteins with *p* values < 0.05 and fold changes > 1.2 or <0.83 were considered as significantly differentially abundant proteins (DAPs).

### 2.7. Functional Annotation of the Proteins

Functional annotation of the proteins was conducted using the Blast2GO program against NCBI's nonredundant protein database to assign Gene Ontology (GO) terms. The Kyoto Encyclopedia of Genes and Genomes (KEGG) database (http://www.genome.jp/kegg/) and the Clusters of Orthologous Groups (COG) database (http://www.ncbi.nlm.nih.gov/COG/) were used to classify and group the identified proteins.

### 2.8. Quantitative Real-Time PCR Analysis

The four samples PF, PFI, PFI-15, and PFI-75 that were stored at −80°C were used to extract total RNAs by Trizol reagent. After purification with DNase I, the concentrated RNAs were reverse transcribed into cDNAs with a GoScript™ Reverse Transcription System. The 18S primers were Forward, 5′-ACATAGTAAGGATTGACAGA-3′, and Reverse, 5′-TAACGGAATTAACCAGACA-3′. We randomly selected 14 DAPs and obtained the genes encoding them by BLAST searches. The primers that were designed for the 14 genes are listed in Table 1. The PCR reactions were performed in 20 *μ*L final volume, including 2 *μ*L cDNAs, 10 *μ*L SYBR, and 6.4 *μ*L ddH2O with the following thermal cycle: 95°C for 3 min, followed by 40 cycles of 95°C for 10 s, and 57°C for 20 s. The relative expression of the 14 genes were calculated by the 2^−ΔΔCT^ relative quantization method.

## 3. Results

### 3.1. Morphological Analysis and Phytoplasma Detection in *P. fortunei*

**Figure 1:**
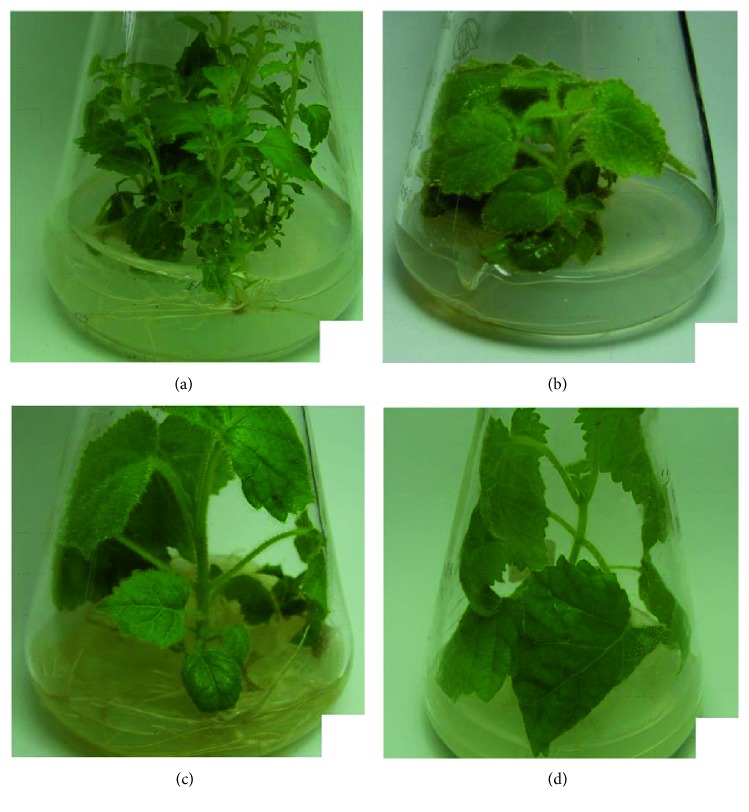
Morphology of *P. fortunei* seedlings treated with dimethyl sulfate (DMS). (a) Morphology of PFI. (b) Morphology of 15 mg·L^−1^ DMS-treated PFI. (c) Morphology of 75 mg·L^−1^ DMS-treated PFI. (d) Morphology of PF.

**Figure 2:**
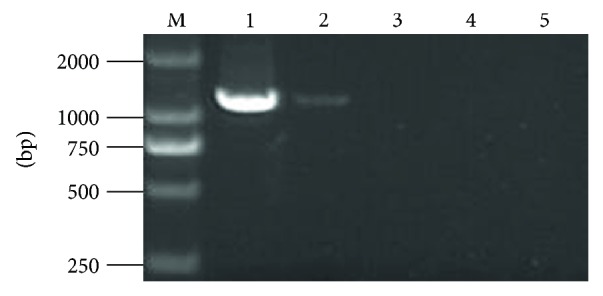
Phytoplasma 16S rDNA amplication in DMS-treated *P. fortunei*. (M) DNA marker. (1) PFI. (2) PFI-15. (3) PFI-75. (4) PF. (5) ddH_2_O.

*P. fortunei* seedlings infected with PaWB were treated with different concentration of DMS. Through observation, we found that the rooting rate and other morphologic changes were distinctly different in PaWB-infected *P. fortunei* with DMS treatment (Table 2 and Figure 1). On the 10th day of treatment, the rooting rate was 96.67% in PFI, 86.67% in PFI-15, and 0% in PFI-75; on the 20th day, the rooting rate was 100% in both PFI and PFI-15 and 40% in PFI-75; and on the 30th day, the rooting rate of PFI-75 was 60%. Thus, the rooting rates decreased with increased DMS concentrations. Morphologically, the PFI seedlings were characterized by witches' broom, shortened internodes, swollen terminal buds, yellowing of leaves, and smaller leaves without chaeta. After the 15 and 75 mg·L^−1^ DMS treatments, the seedlings recovered a healthy morphology, such as normal internodes and terminal buds, no axillary buds, and green leaves with chaeta. In addition, fragments (1.24 kb in length) of the 16S rDNA from the PaWB phytoplasma [30] were found to be expressed in the PFI and PFI-15 seedlings, but not in the PF and PFI-75 seedlings by nested PCR (Figure 2). In our previous studies [11], we found that there were no changes in the DNA sequences at the simple sequence repeat level among the PaWB-infected Paulownia and recovered PaWB-infected Paulownia that had healthy morphology due to DMS treatment. The global DNA methylation level of diseased seedlings was lower than that of healthy seedlings, and the global DNA methylation level of recovered healthy diseased seedlings due to oxytetracycline treatment was higher than that of untreated diseased seedlings [31]. Consequently, it might be inferred that DMS may enhance DNA methylation in PaWB-infected seedlings allowing them to recover and become healthy. Further, DMS may restrain not only phytoplasma growth and division but also, to an extent, the regular growth of seedlings.

### 3.2. Statistical Analysis and Identification of Proteins in the *P. fortunei* Proteome

**Figure 3:**
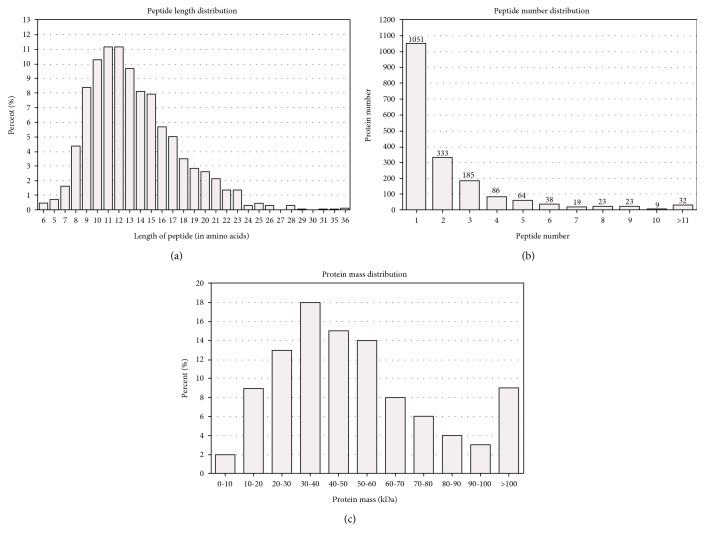
Statistics of basic information of proteins. (a) Percentage of different length of peptide in all peptides. (b) Distribution of matched peptide number in identified proteins. (c) Distribution of relative molecular mass of identified proteins.

Through the application of iTRAQ technology, we obtained 458,154 total spectra for the two biological replicates using a mass spectrometer TripleTOF 5600 (Table 3). After Mascot analysis and data filtering to eliminate low-scoring spectra, 18,216 spectra, which represented approximately 3.98% of the total spectra, were matched. Subsequently, 10,870 unique spectra were detected and a total of 2969 proteins were identified for further analysis (Supplementary Table 1). The majority of peptides were from nine to 15 amino acids long and peptides with 11 and 12 amino acids accounting for approximately 22% of all peptides (Figure 3(a)). The number of identified proteins was highest when only one peptide matched and decreased as the numbers of peptides that matched the proteins increased (Figure 3(b)). Proteins with masses of 30–40 kDa were the most abundant, followed by proteins of 40–50 kDa and 50–60 kDa (Figure 3(c)). The producibility of the proteomic analysis showed that the proteome results were reliable (Supplementary Figure 1).

Functional classification was performed based on the three main GO categories, biological process, cellular component, and molecular function (Supplementary Figure 2). Under cellular component, the largest numbers of proteins were annotated as involved in both cell (20.29%) and cell part (20.29%). Under molecular function, the largest number of proteins was annotated as involved in binding (42.44%). Under biological process, the largest number of proteins was annotated as involved in metabolic process (18.26%). Together, these two terms accounted for nearly half the annotations assigned under molecular function, which implied that the 2969 identified proteins mainly played prominent roles in binding and catalytic activity.

The function classification based on the COG database assigned the identified proteins to 23 categories (Supplementary Figure 3). Among them, “posttranslational modification, protein turnover, chaperones” was the largest category with 379 proteins, followed by “energy production and conversion,” “general function prediction only,” and “carbohydrate transport and metabolism,” all of which contained more than 300 proteins. “Cell motility” was the smallest group with only two proteins.

### 3.3. Analysis of Differentially Abundant Proteins in *P. fortunei*

Proteins with fold changes > 1.2 or <0.83 and *p* values < 0.05 were considered to be differentially abundant. We detected 106 DAPs in the PF versus PFI-75 (44 more abundant and 62 less abundant), 82 in PFI versus PFI-15 (44 more abundant and 38 less abundant), 64 in PFI-15 versus PFI-75 (29 more abundant and 35 less abundant), and 104 in PF versus PFI (37 more abundant and 67 less abundant). The PFI versus PFI-15 comparison was the only one in which the number of more abundant proteins was higher than the number of less abundant proteins.

The 104 DAPs that were identified in PF versus PFI may help to reveal the response of *P. fortunei* to phytoplasma infection. Based on the functional analysis of these DAPs, 39 KEGG pathways responsive to phytoplasma infection were detected, including the highly enriched “photosynthesis,” “photosynthesis-antenna proteins,” and “ribosome” pathways (Supplementary Table 2). The GO functional analysis revealed 55 biological process terms, 30 cellular component terms, and 12 molecular function terms that were significantly enriched (Supplementary Table 3).

To analyze the response of PaWB-infected *P. fortunei* to DMS treatment, we compared the DAPs in the PFI versus PFI-15 and PFI-15 versus PFI-75 comparisons and chose the 32 DAPs that were common in these two comparisons as the DMS-responsive DAPs. The annotations indicated that the 32 DAPs were related to photosynthesis, protein synthesis, metabolism, and nitrogen metabolism (Supplementary Table 4).

To detect proteins that were most likely to be associated with PaWB, we considered that the accumulated level of a protein in PF versus PFI would be opposite to its accumulated levels in the PFI versus PFI-15 and PFI-15 versus PFI-75 comparisons. Therefore, we selected proteins that were more abundant in PF versus PFI and less abundant in one or both of the PFI versus PFI-15 and PFI-15 versus PFI-75 comparisons, as well as the proteins that were less abundant in PF versus PFI and more abundant in one or both of the PFI versus PFI-15 and PFI-15 versus PFI-75 comparisons. Based on these criteria, we obtained 27 predicted PaWB-related proteins (three were differentially accumulated in all three comparisons and 24 were differentially accumulated in two of the comparisons) for the enrichment analysis (Supplementary Table 5).

**Figure 4:**
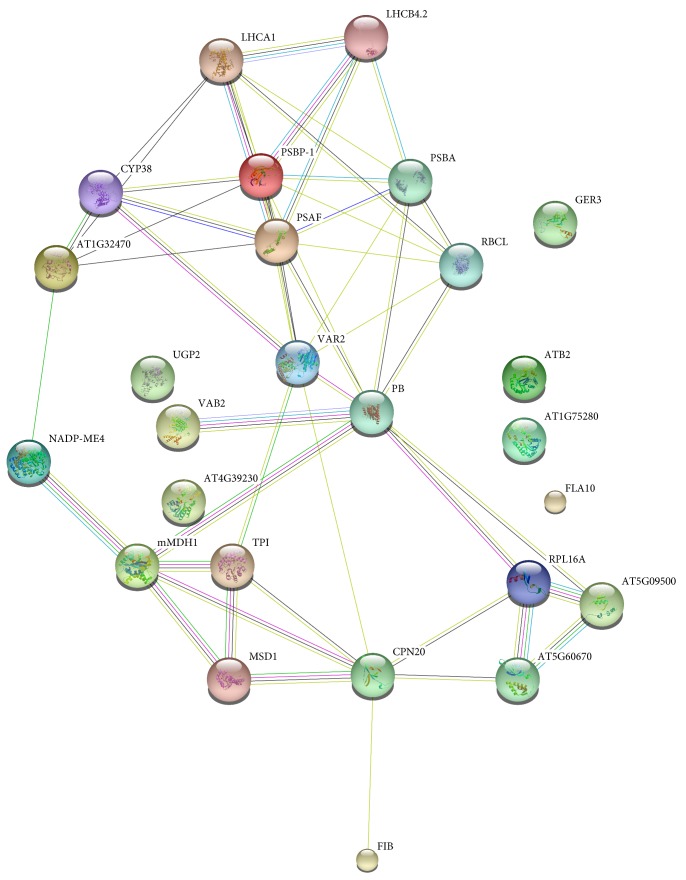
Functional networks of the identified PaWB-related proteins. Different line colors represent the types of evidence used in predicting the associations: gene fusion (red), neighborhood (green), cooccurrence across genomes (blue), coexpression (black), experimental (purple), association in curated databases (light blue), text mining (yellow), and homology (light purple).

The GO and KEGG pathway analyses of the 27 selected proteins revealed possible interactions between the phytoplasma and the PaWB-related proteins. The GO enrichment analysis (Supplementary Table 6) identified 12 terms that were significantly enriched (*p* value < 0.05) under biological process with “response to abiotic stimulus” accounting for the highest number of proteins (11). Under cellular component, 16 terms were significantly enriched with “thylakoid” accounting for the highest number of proteins (11), and under molecular function, only four terms were significantly enriched with “metal ion binding” and “cation binding” both accounting for the highest numbers of proteins (11). The KEGG pathway analysis mapped the 27 DAPs to 23 pathways, with metabolic pathways accounting for the highest numbers of proteins (Supplementary Table 7). To better understand the functions and interactions of the identified 27 PaWB-related DAPs, a protein-protein interaction network analysis was performed with the online analysis tool STRING1 (version 10, http://string-db.org). Because the protein annotations in the NCBI plant database are based on different organisms, the 27 proteins were searched against the *Arabidopsis thaliana* protein database to obtain annotated protein entries for the network analysis (Figure 4() and Supplementary Table 5). The results suggest that PB (gi|13236786, ATP synthase subunit beta), LHCB4.2 (gi|445116, chlorophyll a-b binding protein CP29.2), mMDH1 (gi|21388550, malate dehydrogenase 1), and RPBL16A (gi|1173055, 60S ribosomal protein L11-1) are the central points in the network and play essential roles in response to PaWB.

### 3.4. Expression Levels of Genes Encoding 14 Selected DAPs by qRT-PCR

**Figure 5:**
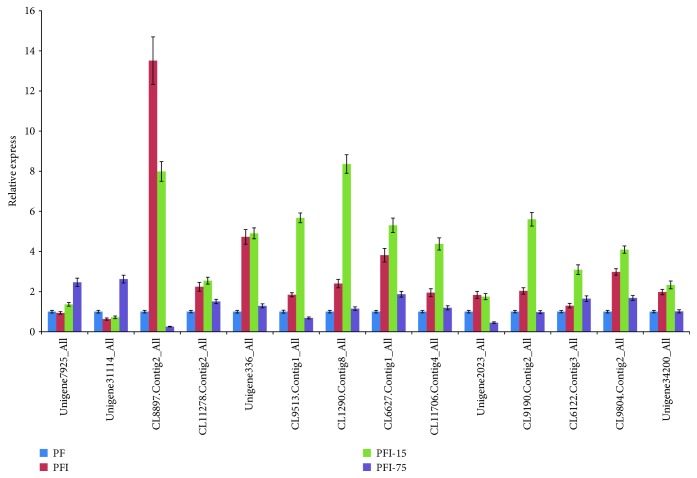
Relative expression levels of the mRNA in *P. fortunei* by qRT-PCR. PF: healthy *P. fortunei*. Unigene7925_All: cytosolic NADP-malic enzyme; Unigene31114_All: ribulose-1, 5-bisphosphate carboxylase/oxygenase; CL8897.Contig2_All: germin-like protein; CL11278.Contig2_All: ATP synthase; Unigene336_All: anganese superoxide dismutase; CL9513.Contig1_All: chloroplast pigment-binding protein CP29; CL1290.Contig8_All: auxin-induced protein PCNT115-like; CL6627.Contig1_All: mitochondrial NAD-dependent malate dehydrogenase; CL11706.Contig4_All: peptidyl-prolyl cis-trans isomerase; Unigene2023_All: fasciclin-like arabinogalactan protein 10-like; CL9190.Contig2_All: chlorophyll a-b binding protein; CL6122.Contig3_All: ATP-dependent zinc metalloprotease FTSH 2; CL9804.Contig2_All: V-type proton ATPase subunit B2 OS = *Arabidopsis thaliana*; Unigene34200_All: UDP-glucose pyrophosphorylase. The 18S rRNA was acted as an internal reference gene for normalization. The normalized mRNA transcript levels were arbitrarily set to 1 in PF. Standard errors of the mean are represented by the error bars.

To add supplementary information to the iTRAQ analysis, we examined the transcript expression levels of the genes encoding 14 randomly selected proteins by qRT-PCR (Figure 5). Among the four comparisons, only CL6122.Contig3_All displayed the same expression trend as the corresponding DAP in the iTRAQ data in PF versus PFI, PF versus PFI-75, and PFI-15 versus PFI-75, but not in PFI versus PFI-15. At the transcript expression levels, CL8897.Contig2_All, CL11278.Contig2_All, and CL9804.Contig2_All were consistent with the corresponding DAPs in the iTRAQ data in two of the four comparisons, whereas the expression levels of Unigene7925_All, Unigene31114_All, and Unigene34200_All showed opposite trends with the corresponding DAPs in the iTRAQ data. These results suggested that gene expression at the transcriptome level did not necessarily reflect the abundance of the encoded proteins encoded, likely because posttranscriptional processing and posttranslational modification are crucial processes in plants.

## 4. Discussion

Proteomics has been used in plants to help understand their response to stressful conditions. In this study, we identified 2969 proteins that will form a database for further studies. In addition, 104 DAPs were identified between healthy and infected seedlings. In other studies of phytoplasma-infected plants, such as lime [32], mulberry [4], tobacco [19], and grape [33], 990, 500, 1466, and 576 proteins, respectively, were identified and 448, 37, 330, and 33 DAPs were detected between healthy and infected plants. The differences in protein numbers arise mainly from the proteomic approaches used in each study. The results suggest that label-free (lime) shotgun proteomics may reveal more DAPs, whereas label-based (Paulownia and tobacco) proteomics may identify more proteins. The iTRAQ technology that we used in our study has been proven to be a powerful method to investigate proteomic changes, because many proteins can be identified simultaneously and proteomic changes can be measured with high sensitivity. In four previous studies [4, 19, 32, 33], we found that the Paulownia proteins that responded to phytoplasma infection mostly were related to photosynthesis, protein metabolism, signal transduction, and cell defense, and “photosynthesis” and “ribosome” were enriched pathway, “photosynthesis,” and “ribosome” were also enriched in this study, suggesting common responses of to phytoplasma infection. In our previous study [11], we also found that DMS treatment allowed the morphology of infected plants to recover and led to the elimination of the phytoplasmas in PaWB-infected plants. Although DMS could induce methylation of adenine [34], it did not change the DNA sequences at the simple sequence repeat level. We also found that oxytetracycline treatment is similar to DMS treatment, as both of these treatments could make the PaWB-infected Paulownia recover to a healthy morphology. It has been previously demonstrated that oxytetracycline treatment can influence the global DNA methylation level of PaWB-infected Paulownia [31], with possible effects on plant development and other processes [35]. In the light of these observations, the DMS treatment might influence the DNA methylation level in infected Paulownia, thereby influencing the processes which could regulate plant morphogenesis, finally resulting in morphological changes. In this study, we treated PFI seedlings with DMS and detected DMS-responsive DAPs. Many of these DAPs were photosynthetic proteins that were also considered to be phytoplasma responsive. If this is the effecter that causes the PaWB symptoms of infected seedlings to recover to normal, a future in-depth study is needed.

The DMS treatment helped us to identify 27 PaWB-related proteins that we classified into four groups: photosynthesis-related (LHCB4.2, LHCA1, CYP38, AT1G32470, PSBP-1, PSAF, PSBA, RBCL,VAR2 PB, VAB2, and FIB), energy-related (NADP-ME4, Mmdh1, TPI, MSD1, and CPN20), ribosome-related (RPL16A, AT5G09500, and AT5G60670), and individual proteins (UGP2 (gi|183397343, UDP-glucose pyrophosphorylase 2), AT1G75280 (gi|4731376, Isoflavone reductase-P3), AT4G39230 (gi|359475114, NmrA-like negative transcriptional regulator family protein), GER3 (gi|222051768, Germin 3), ATB2 (gi|356526627, Encodes ATB2), FLA10 (gi|224130034, FASCICLIN-like arabinogalactan-protein 10)). Phytoplasmas need to obtain metabolites from the host plants because they lack several metabolic enzymes involved in the pentose phosphate pathway and the F1F0-type ATP-synthase subunit [35, 36], and phytoplasmas secrete virulence factors to suppress the growth and development of host plants through the Sec protein translocation system [37–39]. Photosynthesis-, ribosome-, and energy-related proteins were already known to be associated with phytoplasmas infection [4, 19, 32, 33]; however, the relationship between the individual proteins and PaWB is yet to be established.

UDP-glucose pyrophosphorylase (UGP2, gi|183397343) catalyzes the reversible production of UDP-glucose and pyrophosphate from glucose-1-phosphate and UTP and is a key enzyme for carbohydrate metabolism. UGP2 is associated with the metabolism of glucose, which can be used by phytoplasmas as a source of energy [40], and may be involved in the Paulownia-phytoplasma interaction. ATB2 (gi|356526627, Encodes ATB2) is a NAD(P)-linked oxidoreductase superfamily protein, which has been identified as a salt-response protein [41].

Flavonoids are significant secondary metabolites that regulate auxin transport, seed germination, signaling pathways with symbiotic microorganisms, and resistance [42]. Flavonoids have been classified into six categories: flavones, flavonols, flavanones, isoflavones, flavanols, and anthocyanin [43]. Conserved enzymes in the biosynthesis pathway of flavonoids are mostly divided into three groups: ketoglutarate-dependent dioxygenases, including flavanone 3-hydroxylase, flavone synthase, and leucoanthocyanidin dioxygenase; NADPH-dependent reductases, including dihydroflavonol-4-reductase, leucoanthocyanidin reductase, and isoflavone reductase; and cytochrome P450 hydroxylases, including flavonoid 3′-hydroxylase, flavonoid 3′,5′-hydroxylase, and isoflavone synthase. AT1G75280 (IFR, gi|4731376) belongs to the NADPH-dependent reductase family. The At4g39230 gene encodes a protein sequence that is similar to phenylcoumaran benzylic ether reductase and has been found to influence the levels of flavonoids [44]. Therefore, the protein annotated as similar to AT4G39230 (gi|359475114, NmrA-like negative transcriptional regulator family protein) in our results may be related to flavonoid metabolism. Flavonoid metabolism and flavonoid biosynthesis genes are known to be activated as part of plant defense responses after infection [45]. The flavonoid metabolism-related proteins (AT1G75280 and AT4G39230) that we detected showed higher abundance in PFI compared with PF, implying they may be PaWB-responsive proteins.

Germin-like proteins (GLPs) are extracellular glucoproteins that belong to the pathogenesis-related protein family. Germin-like proteins act as enzymes (oxalate oxidase and SOD), receptors, and structural proteins in response to biotic and abiotic stresses and have been categorized into three subfamilies [46]. Germin 3 (gi|222051768, auxin-binding protein 19), which was detected among the DAPs in this study, belongs to subfamily 3, which contains regulatory proteins related to auxin metabolism [47]. Auxin-binding proteins (ABPs) serve as auxin receptors and are located in the membranes of endoplasmic reticulum, vacuole, and cytoplasm. Ohmiya et al. first isolated ABP19 from shoot apices in peach and found that it bound specifically with auxin [48]. Subsequently, ABP19 has been found on the cell wall of palisade parenchyma cells and in spongy parenchyma cells in leaves [49]. During auxin signal transduction, auxins combine with ABPs on the membrane and then activate G-proteins, which induce the intracellular signal transduction of auxin, finally causing a variety of physiological and biochemical responses in plants. In our study, ABP19 was found to be more abundant in the PF versus PFI and PF versus FPI-75 comparisons and less abundant in the PFI versus PFI-15 comparison. The higher accumulation of ABP19 in PFI compared with PF may be why PF released more ABP19 to not only enhance the efficiency of auxin signal transduction in response to the decrease of auxin but also to strengthen the cell wall to guard against phytoplasma attack.

In summary, we analyzed the physiological characteristics and determined the abundances of proteins using iTRAQ labeling coupled with LC-MS/MS in PF, PFI, PFI-15, and PFI-75 seedlings. We found that DMS was able to eliminate PaWB phytoplasmas in *P. fortunei*. In total, 2969 proteins were identified, and 104 phytoplasma infection-responsive and 32 DMS-responsive DAPs were found. In addition, 27 PaWB-related proteins were discovered, and their protein-protein interactions were analyzed. Our results provide new insights for further analyses of the molecular mechanisms of how PaWB phytoplasma influences phytohormones in *P. fortunei*.

## Supplementary Material

The information of supplementary materials are as follows: Supplementary Figure 1 The repeatability of two replicates. A: PF *vs.* PFI, B: PFI *vs.* PFI-15, C: PFI-15 *vs.* PFI-75, D: PF *vs.* PFI-75. The ratios of protein abundances for each protein in each comparison between biological replicates were calculated, and the “delta, error” in the absciss are presents the difference from the expected ratio of 1. Supplementary Figure 2 Gene Ontology (GO) analysis of proteins identified in *P. fortunei.* (A) Cellular component of GO. (B) Molecular function of GO. (C) Biological process of GO. Supplementary Figure 3 Cluster of Orthologous Groups (COG) function classification of proteins identified in *P. fortunei.* Supplementary Table 1: All proteins identified by iTRAQ in *P. fortunei.* Supplementary Table 2: KEGG annotation of the DAPs in PF vs. PFI. Supplementary table 3: GO analyses of the DAPs in PF vs. PFI. Supplementary table 4: Annotations of the 32 DMS-responsive DAPs. Supplementary Table 5: Statistics of 27 predicted PaWB-related proteins in *P. fortunei.* Supplementary Table 6: TOP 10 GO function analysis of DAPs in *P. fortunei.* Supplementary Table 7: KEGG pathway analysis of DAPs in *P. fortunei.* Supplemental Protocol 1: The program of SCX chromatography. Supplemental Protocol 2: The program of LC-MS/MS analysis. Supplemental Protocol 3: The parameters of Proteome Discoverer software.

## Figures and Tables

**Table 1:** Primers of 14 genes used for qRT-PCR analysis.

Unigene ID	Sense primer	Antisense primer
Unigene7925_All	AACACAGGAACATTATGAT	CTCTCAGCATACTTAACC
Unigene31114_All	GGTGAATGTGAAGAAGTA	GGAGTTCCTATCGTAATG
CL8897.Contig2_All	TCATCTCTTCTGCTAACG	TTACCACCTCCATTGATT
CL11278.Contig2_All	AGAGCCTATTGATGAGAG	ACCTTGATACCAGTTACA
Unigene336_All	ATATGTTCTTCAGGTAGTC	TTCCATTACTTGGTATTGA
CL9513.Contig1_All	GAACCAAGAAATCTCCTC	AGCCATCCAGATATTCAG
CL1290.Contig8_All	CTCATCGGATTGATACTC	CTCTGATAGACCAACATAT
CL6627.Contig1_All	TCCTCTCGTCATATACTC	CCAATGCTCTTGTCAATA
CL11706.Contig4_All	AAGAGGATATGGTTGATG	ACAATACGGAATACACAT
Unigene2023_All	CTATGGCTGACTGGTATA	CAACATTACGGCTTTACT
CL9190.Contig2_All	CTCAAAGTCAAGGAAATC	ATAATGATGTCTCCAATGT
CL6122.Contig3_All	GATTCAGATGTATCACTTG	CAGCAACTATCCTATCAA
CL9804.Contig2_All	AAGAGCATCAGCATAAGA	CCAACAATAGAACGCATTA
Unigene34200_All	GGTACTCTGATCTCTTATG	TGAACTTCTCTATTGACTTA

**Table 2:** Rooting rates and morphologic changes in PaWB-infected *P. fortunei* with DMS treatment.

Samples	Concentrations (mg·L^−1^)	Rooting rates at various days (%)			Axillary buds	Internodes and leaves	Terminal bud growth
		10 d	20 d	30 d			
PFI	0	96.67	100	100	With	Shortened internodes and small and yellow leaves without chaeta	Swelling
PFI-15	15	86.67	100	100	Without	Normal internodes and green leaves with chaeta	Normal
PFI-75	75	0	40	60	Without	Normal internodes and green leaves with chaeta	Normal

PFI: PaWB-infected *P. fortunei*; PFI-15: 15 mg·L^−1^ DMS-treated PFI; PFI-75: 75 mg·L^−1^ DMS-treated PFI.

**Table 3:** Statistics of proteins identified by iTRAQ.

Group name	Total spectra	Spectra	Unique spectra	Peptide	Unique peptide	Protein
Repeat 1	232,791	9037	5296	3030	2194	1863
Repeat 2	225,363	9179	5574	3455	2519	2121
Combined	458,154	18,216	10,870	—	—	2969
