# Supplementary figures and images for: Comparative Proteomic Analysis of Paulownia fortunei Response to Phytoplasma Infection with Dimethyl Sulfate Treatment
Source: Int J Genomics. 2017 Sep 5;2017:6542075. doi: 10.1155/2017/6542075 (PMC5605944; doi:10.1155/2017/6542075)

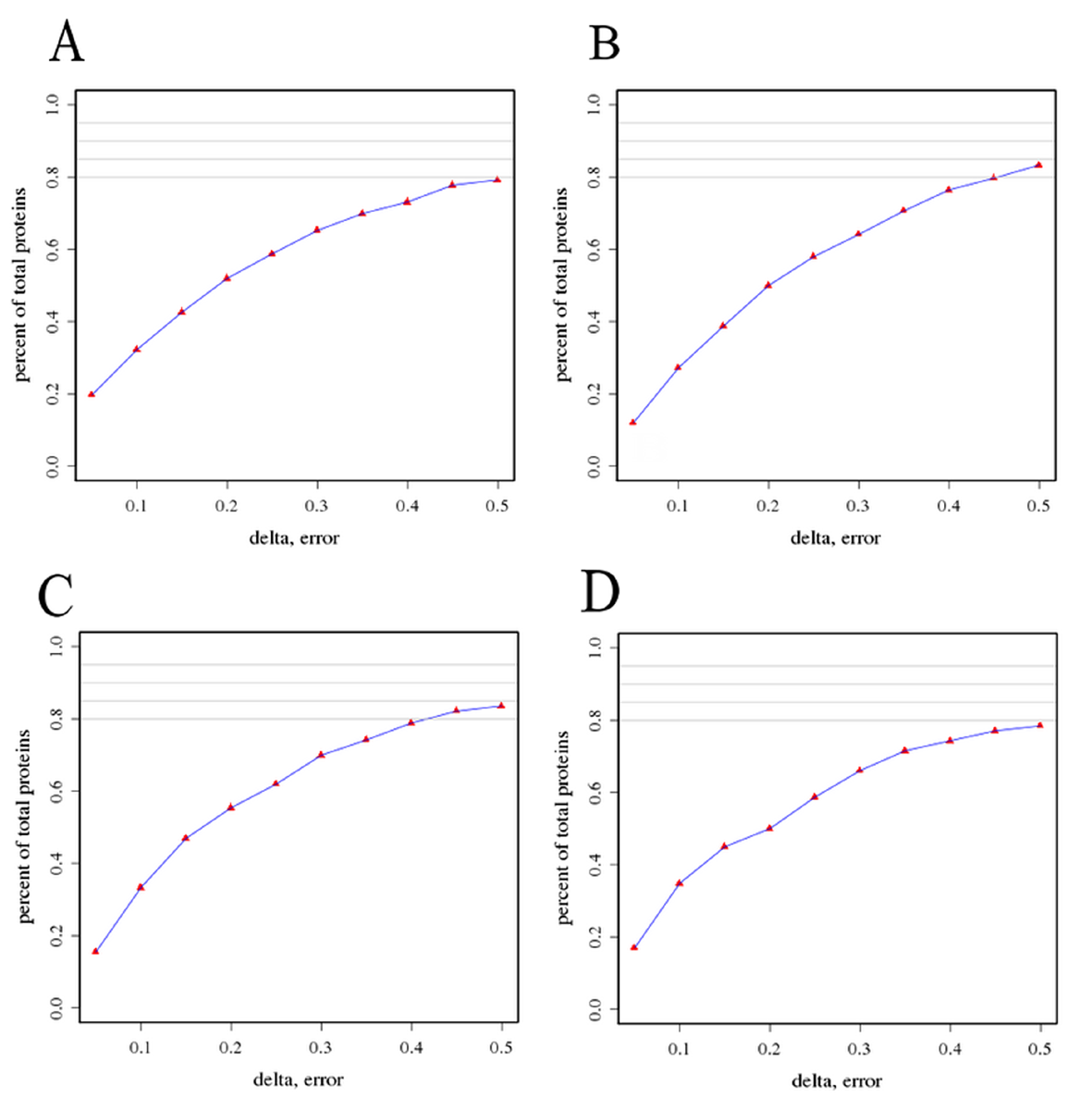

Supplement: Supplementary file 2 [file 6542075.f2.tif]

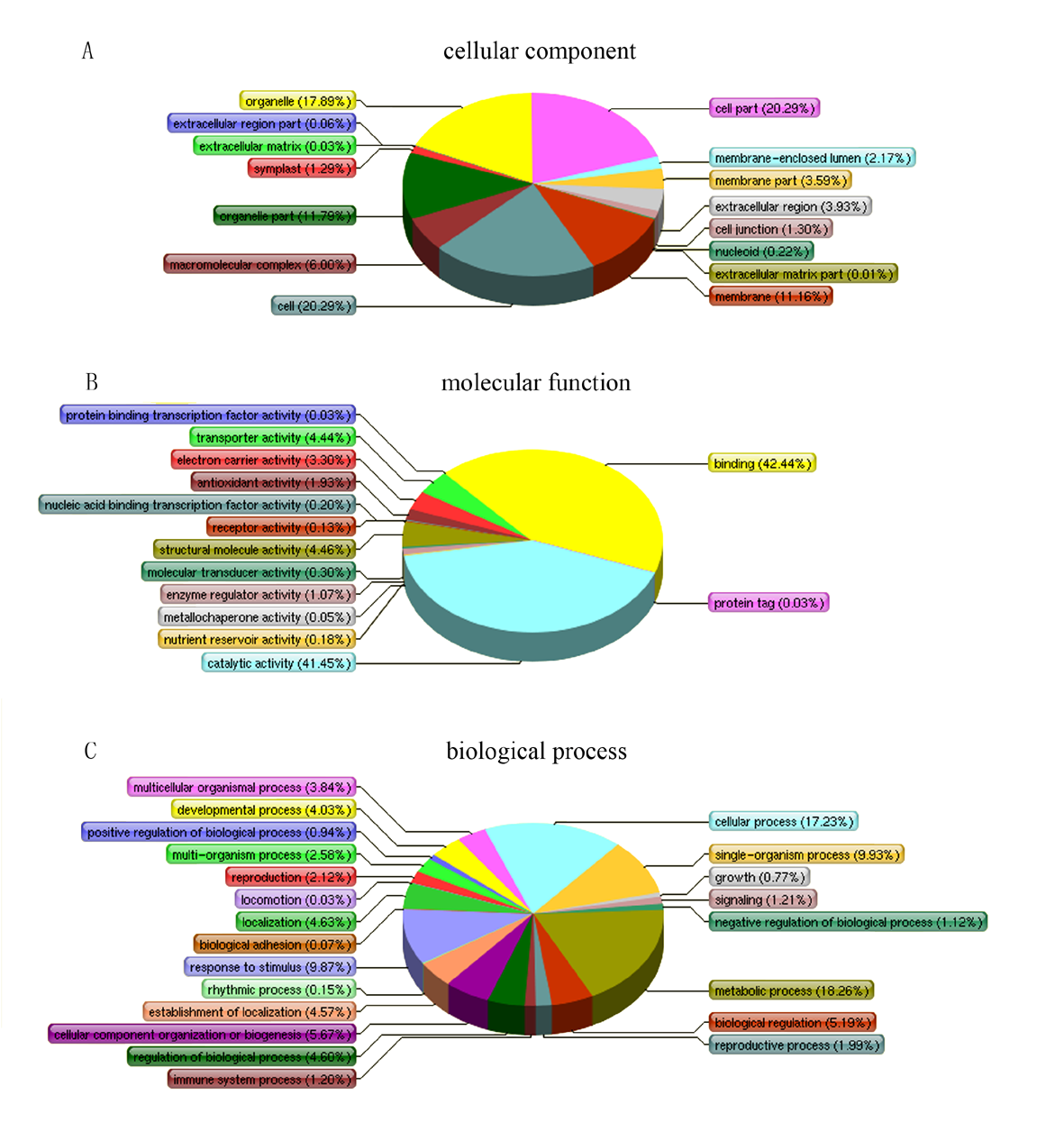

Supplement: Supplementary file 4 [file 6542075.f4.tif]

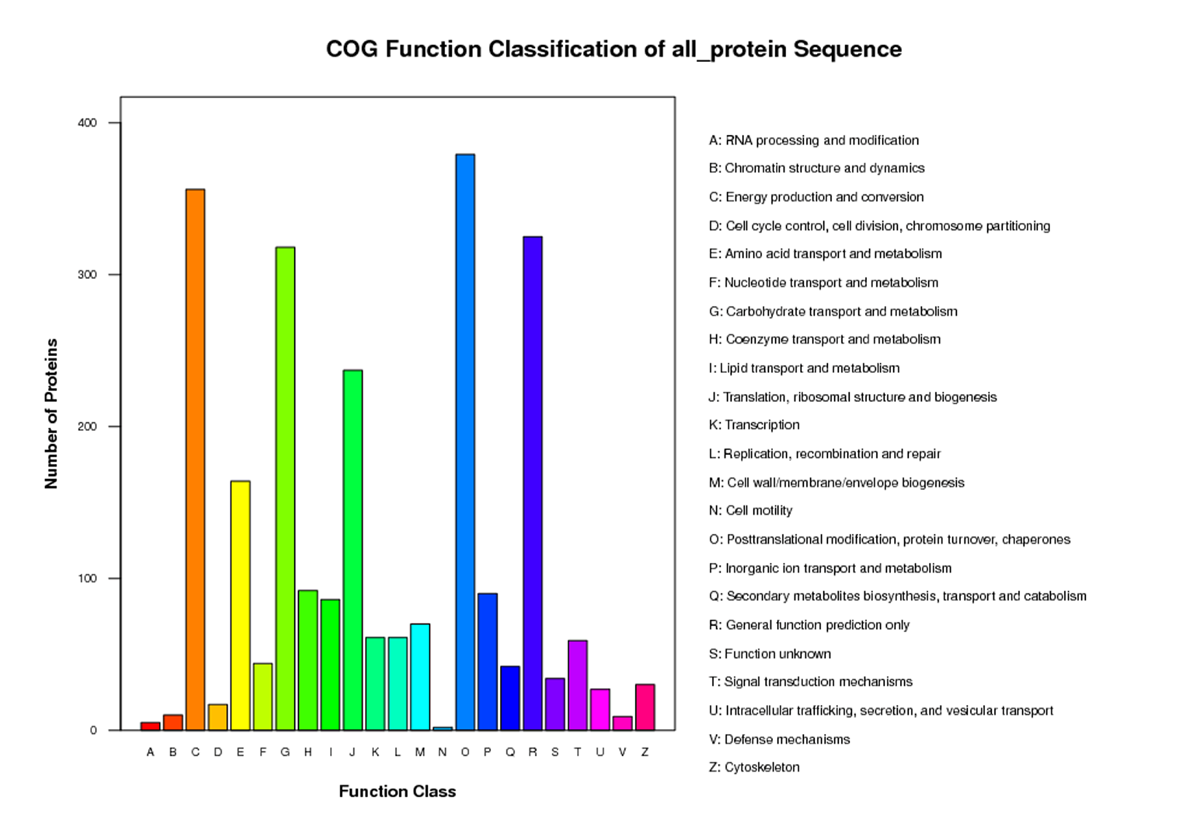

Supplement: Supplementary file 6 [file 6542075.f6.tif]
