# Supplementary material for: Comparative Proteomic Analysis of Paulownia fortunei Response to Phytoplasma Infection with Dimethyl Sulfate Treatment
Source: Int J Genomics. 2017 Sep 5;2017:6542075. doi: 10.1155/2017/6542075 (PMC5605944; doi:10.1155/2017/6542075)
Supplement: Supplementary file 12 [file 6542075.f12.docx]

Supplementary Table 6: TOP 10 GO function analysis of DAPs in *P. fortunei*.

| Ontology | Class | DAP-Number |
| --- | --- | --- |
| Cellular component | chloroplast thylakoid membrane | 10 |
|  | plastid thylakoid membrane | 10 |
|  | thylakoid membrane | 10 |
|  | photosynthetic membrane | 10 |
|  | thylakoid part | 10 |
|  | chloroplast thylakoid | 10 |
|  | plastid thylakoid | 10 |
|  | organelle subcompartment | 10 |
|  | membrane | 18 |
|  | plastoglobule | 4 |
| Molecular function | metal ion binding | 12 |
|  | cation binding | 12 |
|  | malate dehydrogenase activity | 2 |
|  | transition metal ion binding | 7 |
|  | 2-alkenal reductase [NAD(P)] activity | 2 |
|  | structural molecule activity | 4 |
|  | ion binding | 14 |
|  | ATPase activity, coupled | 3 |
|  | structural constituent of ribosome | 3 |
|  | oxidoreductase activity, acting on the CH-CH group of donors, NAD or NADP as acceptor | 2 |
| Biological process | regulation of cellular component size | 2 |
|  | regulation of anatomical structure size | 2 |
|  | photoinhibition | 2 |
|  | negative regulation of photosynthesis, light reaction | 2 |
|  | regulation of photosynthesis, light reaction | 2 |
|  | regulation of generation of precursor metabolites and energy | 2 |
|  | response to abiotic stimulus | 11 |
|  | regulation of photosynthesis | 2 |
|  | negative regulation of biological process | 4 |
|  | malate metabolic process | 2 |
