# Supplementary material for: Comparative Proteomic Analysis of Paulownia fortunei Response to Phytoplasma Infection with Dimethyl Sulfate Treatment
Source: Int J Genomics. 2017 Sep 5;2017:6542075. doi: 10.1155/2017/6542075 (PMC5605944; doi:10.1155/2017/6542075)
Supplement: Supplementary file 13 [file 6542075.f13.docx]

Supplementary Table 7: KEGG pathway analysis of DAPs in *P. fortunei*.

| Pathway | DAPs with pathway annotation | *p*-Value | Pathway ID |
| --- | --- | --- | --- |
| Flavonoid biosynthesis | 2 (8.7%) | 0.004882384 | ko00941 |
| Propanoate metabolism | 2 (8.7%) | 0.03030097 | ko00640 |
| Carbon fixation in photosynthetic organisms | 5 (21.74%) | 0.03326022 | ko00710 |
| Glyoxylate and dicarboxylate metabolism | 4 (17.39%) | 0.03793777 | ko00630 |
| Photosynthesis - antenna proteins | 2 (8.7%) | 0.0481484 | ko00196 |
| Inositol phosphate metabolism | 1 (4.35%) | 0.06733825 | ko00562 |
| Photosynthesis | 4 (17.39%) | 0.1515576 | ko00195 |
| Vitamin B6 metabolism | 1 (4.35%) | 0.1560569 | ko00750 |
| Ribosome | 3 (13.04%) | 0.1770405 | ko03010 |
| Metabolic pathways | 13 (56.52%) | 0.2094613 | ko01100 |
| Biosynthesis of secondary metabolites | 7 (30.43%) | 0.2167547 | ko01110 |
| Pentose and glucuronate interconversions | 1 (4.35%) | 0.2289522 | ko00040 |
| Galactose metabolism | 1 (4.35%) | 0.2518827 | ko00052 |
| Pyruvate metabolism | 2 (8.7%) | 0.2751587 | ko00620 |
| Phagosome | 1 (4.35%) | 0.3098857 | ko04145 |
| Peroxisome | 1 (4.35%) | 0.3570869 | ko04146 |
| Oxidative phosphorylation | 2 (8.7%) | 0.3898975 | ko00190 |
| Fructose and mannose metabolism | 1 (4.35%) | 0.4011909 | ko00051 |
| Ascorbate and aldarate metabolism | 1 (4.35%) | 0.4011909 | ko00053 |
| Starch and sucrose metabolism | 1 (4.35%) | 0.4536567 | ko00500 |
| Amino sugar and nucleotide sugar metabolism | 1 (4.35%) | 0.459207 | ko00520 |
| Citrate cycle (TCA cycle) | 1 (4.35%) | 0.5017085 | ko00020 |
| Glycolysis / Gluconeogenesis | 1 (4.35%) | 0.7465621 | ko00010 |
