# Supplementary material for: Comparative Proteomic Analysis of Paulownia fortunei Response to Phytoplasma Infection with Dimethyl Sulfate Treatment
Source: Int J Genomics. 2017 Sep 5;2017:6542075. doi: 10.1155/2017/6542075 (PMC5605944; doi:10.1155/2017/6542075)
Supplement: Supplementary file 14 [file 6542075.f14.docx]

**The program of SCX chromatography**:

SCX chromatography was performed with a LC-20AB HPLC Pump system (Shimadzu, Kyoto, Japan).

1）**Load**, the iTRAQ labeled peptide mixtures were reconstituted with 4 mL buffer A (25 mM NaH_2_PO4 in 25% ACN, pH 2.7) and loaded onto a 4.6×250 mm Ultremex SCX column containing 5-μm particles (Phenomenex).

2) **Elute**, the peptides were eluted at a flow rate of 1mL/min with a gradient of 5% buffer B (25mM NaH_2_PO4, 1 M KCl in 25% ACN, pH 2.7) for 7 min, 5-60% buffer B for 20 min 60-100% buffer B for 2 min. The system was then maintained at 100% buffer B for 1 min before equilibrating with buffer B for 10 min prior to the next injection.

3)**Collect**, elution was monitored by measuring the absorbance at 214 nm, and fractions were collected every 1 min. The eluted peptides were pooled into 20 fractions, desalted with a Strata X C18 column (Phenomenex) and vacuum-dried.
