# Supplementary material for: Comparative Proteomic Analysis of Paulownia fortunei Response to Phytoplasma Infection with Dimethyl Sulfate Treatment
Source: Int J Genomics. 2017 Sep 5;2017:6542075. doi: 10.1155/2017/6542075 (PMC5605944; doi:10.1155/2017/6542075)
Supplement: Supplementary file 15 [file 6542075.f15.docx]

**The program of LC-MS/MS analysis**

The mass spectroscopy analysis was performed using a TripleTOF 5600 mass spectrometer, coupled with an online micro flow HPLC system. The peptides were separated using a nanobored C18 column with a picofrit nanospray tip (75 μm ID × 15 cm, 5 μm particles) (New Objectives, Wubrun, MA). The separation was performed at a constant flow rate of 20 μL min^−1^, with a splitter to get an effective flow rate of 0.2 μL min^−1^. The mass spectrometer data were acquired in the positive ion mode, with a selected mass range of 300−2000 m/z. Peptides with +2 to +4 charge states were selected for MS/MS. The three most abundantly charged peptides above a count threshold were selected for MS/MS and dynamically excluded for 30 s with ±30 mDa mass tolerance. Smart information-dependent acquisition (IDA) was activated with automatic collision energy and automatic MS/MS accumulation. The fragment intensity multiplier was set to 20, and the maximum accumulation time was 2 s. The peak areas of the iTRAQ reporter ions reflect the relative abundance of the proteins in the samples. For peptide identification, a Triple TOF 5600 mass spectrometer used in this study has high mass accuracy (less than 2 ppm). Other identification parameters used included: fragment mass tolerance: ±0.1 Da; mass values: monoisotopic; variable modifications: Gln->pyro-Glu (N-term Q), oxidation (M), iTRAQ8plex (Y); peptide mass tolerance: 0.05 Da; max. missed cleavages, 1; fixed modifications: carbamidomethyl (C), iTRAQ8plex (N-term), iTRAQ8plex (K); Other parameters: default.
