# Supplementary material for: Comparative Proteomic Analysis of Paulownia fortunei Response to Phytoplasma Infection with Dimethyl Sulfate Treatment
Source: Int J Genomics. 2017 Sep 5;2017:6542075. doi: 10.1155/2017/6542075 (PMC5605944; doi:10.1155/2017/6542075)
Supplement: Supplementary file 16 [file 6542075.f16.docx]

**The parameters of Proteome Discoverer software**

The MS data were processed using Proteome Discoverer software (Version 1.2.0.208) (Thermo Scientific) to generate a peak list. The default parameters of Proteome Discoverer software (Version 1.2.0.208) were used. In detail, they were : for precursor selection, we used the MS1 precursor. For spectrum properties filter: lower rt limit, 0; upper rt limit, 0; lowest charge state, 0; highest charge state, 0; min. precursor mass, 0; max. precursor mass, 300 Da; total intensity threshold, 10 000 Da; minimum peak count, 1. For scan event filters: mass analyzer, FTMS; Ms order, MS2; activation type, HCD; scan type, full; ionization source, nanospray; polarity mode, +. For peak filters: S/N threshold (FT-only), 0. For replacements for unrecognized properties: unrecognized charge, automatic; unrecognized mass analysis, FTMS; unrecognized MS order, MS2; unrecognized activation, HCD; unrecognized polarity Re, +. The data acquisition was performed with Analyst QS 2.0 software (Applied Biosystems/MDS SCIEX).
